# From Protein Misfolding to Extracellular Matrix Disorganisation: Understanding Disease Pathology in Rare Skeletal Dysplasias

**DOI:** 10.3390/ijms262010057

**Published:** 2025-10-15

**Authors:** Ella Patricia Dennis, Michael Darren Briggs

**Affiliations:** Biosciences Institute, International Centre for Life, Newcastle University, Newcastle Upon Tyne NE1 3BZ, UK; michael.briggs@newcastle.ac.uk

**Keywords:** skeletal dysplasia, myopathy, protein misfolding, endoplasmic reticulum stress, unfolded protein response, extracellular matrix disorganisation

## Abstract

Pseudoachondroplasia (PSACH) and multiple epiphyseal dysplasia (MED) are rare, autosomal dominant skeletal dysplasias characterised by disproportionate short stature, joint deformities, and early-onset osteoarthritis. These conditions result from mutations in key cartilage extracellular matrix (ECM) components, including cartilage oligomeric matrix protein (COMP), matrilin-3, and type IX collagen. Although genetically and clinically heterogeneous, PSACH and MED share convergent pathogenic mechanisms. Misfolded mutant ECM proteins are retained within the endoplasmic reticulum (ER) of growth plate chondrocytes, triggering chronic ER stress and impairing chondrocyte proliferation, differentiation, and survival. Moreover, some of the mutant protein is secreted and incorporated into the matrix, leading to altered collagen fibrillogenesis, disrupted proteoglycan distribution, and compromised biomechanical integrity. These alterations extend beyond cartilage, impacting tendons, ligaments, and muscle–tendon junctions, contributing to joint laxity, impaired force transmission, and mild myopathy. This review discusses the structural and functional consequences of ECM disorganisation in PSACH and MED, highlighting its central role in disease progression and emphasising the importance of considering ECM abnormalities when developing therapeutic strategies for rare short stature-associated skeletal disorders.

## 1. Introduction

Genetic skeletal diseases (GSDs) are a diverse group of complex disorders that affect the development, growth, and remodelling of bones. Although individually rare, they have a combined incidence of approximately 1/3500 and primarily present with disproportionate short stature in childhood and early-onset osteoarthritis in young adulthood [1]. To date, over 770 unique conditions resulting from mutations in >550 genes have been described, ranging in severity from mild to severe and lethal forms [1]. These numerous rare bone diseases have been classified into 41 different groups based on radiographic, biochemical (metabolic pathways), and genetic criteria.

GSDs are often chronically debilitating, and as is common with many rare diseases, due to the complexity of disease-causing mechanisms, treatment options to prevent, halt, and/or modify disease progression remain limited. Patient care is currently aimed at supportive and orthopaedic management, providing relief from the symptoms (pain, swelling, stiffness, etc.) rather than treating the disease. Therefore, identifying and understanding common disease mechanisms is essential for the development of new therapies to address an area of unmet clinical need.

## 2. Pseudoachondroplasia and Multiple Epiphyseal Dysplasia

Pseudoachondroplasia (PSACH) and multiple epiphyseal dysplasia (MED) are a group of related short-limbed dwarfisms that affect approximately 1/20,000 children. Due to similar radiographic and ultrastructural findings, PSACH and MED were described as a chondrodysplasia ‘family’ with a spectrum of clinically heterogenous phenotypes characterised by disproportionate short stature and lower limb deformities [2]. In addition to short stature, which is diagnosed in childhood, patients often exhibit ligament laxity and develop progressive joint disease in early adulthood. Not only are PSACH and MED clinically heterogenous, but they are also genetically heterogenous diseases and are caused by autosomal dominant mutations in key cartilage extracellular matrix (ECM) proteins. For example, mild MED (Ribbing type) and more moderate forms of MED (Fairbank type) result from mutations in the cartilage structural proteins type IX collagen, matrilin-3, and cartilage oligomeric matrix protein (COMP), whereas the more severe disease, PSACH, results exclusively from mutations in COMP (Table 1) [3,4,5,6,7].

At present, no validated biochemical biomarkers exist for the clinical diagnosis of PSACH or MED, which relies on radiographic features and genetic testing, the gold standard for diagnosis. However, 10–20% of patients with a clinical diagnosis of MED do not have an identifiable genetic cause. A recent study has shown that plasma COMP levels may serve as a potential biomarker to discriminate between MED genotypes. For example, circulating COMP levels were reduced in patients with *COMP* mutations compared to those without. While these findings are promising, plasma COMP has not been validated as a diagnostic biomarker.

### 2.1. Type IX Collagen

Type IX collagen belongs to the fibril-associated collagens with interrupted triple helices (FACIT) family of collagens, which also includes types XII, XIV, XIX, XXI, and XXII collagen [8]. Type IX collagen is a heterotrimeric molecule composed of three distinct polypeptide α(IX) chains encoded by three separate genes: *COL9A1*, *COL9A2*, and *COL9A3.* All chains comprise three collagenous (COL) domains separated by non-collagenous (NC) domains [9]. Type IX collagen exists in two forms due to the presence of a chondroitin sulphate proteoglycan chain attached to a serine residue in the NC3 domain of the α2(IX) chain. The length and sulphation pattern of this proteoglycan chain are species- and tissue-specific and are relatively short in hyaline cartilage [10]. Further complexity arises from the alternative tissue-specific utilisation of different promoters in *COL9A1*, resulting in two alternatively spliced forms of the α1(IX) chain [11]. For example, the “long” form of α1(IX), which contains a large globular NC4 domain, is found in cartilage, whereas the “short” form lacking this domain is present in early limb buds, the embryonic perinotocordal matrix, as well as the eye (the vitreous, cornea, and retina) [12].

Although type IX collagen is not a fibrillar collagen, this quantitatively minor collagen is attached via lysine-derived covalent cross-links to type II/IX heterotrimeric collagen fibres in growth plate cartilage, articular cartilage, and intervertebral discs [13,14,15]. The regular pattern of type IX collagen along the surface of collagen fibrils, with the COL3 and NC4 domains projecting into the perifibrillar space, indicates that type IX collagen acts as a macromolecular bridge between both collagen fibrils and other matrix proteins [15,16]. It therefore has been deduced that type IX collagen is an important mediator not only for the stabilisation and organisation of collagen network but also in controlling the diameter of collagen fibrils and promoting the anchorage of important cartilage ECM proteins, including matrilin-3 and proteoglycans [17,18].

In 1994, genetic mapping led to the discovery of a novel locus for an unclassified form of MED on the short arm of chromosome 1 (1p32.2-33) [19]. This finding was followed by the identification of heterozygous splice-site mutations in *COL9A2* [20,21]. Subsequently, mutations were also found in *COL9A3* [7,22], with both *COL9A2* and *COL9A3* mutations predominantly affecting splice-donor/acceptor sites in exon 3 and resulting in in-frame deletions within the equivalent regions of the COL3 domain of collagen (α2)IX and collagen (α3)IX (Table 2). A more recent discovery revealed a novel missense mutation (c.Gly35Asp) in exon 2 of *COL9A3,* which does not affect splicing but is hypothesised to disrupt the Pro-Pro-Gly sequence, thus affecting the overall folding and function of collagen IX [23]. Additionally, a single mutation in *COL9A1* has also been linked to MED [5], specifically occurring at the splice acceptor site of exon 8 and leading to the skipping of exon 8 and/or exon 10 and in-frame deletions within the COL3 domain of collagen IX(α1) (Table 2). The exact pathological role of mutant collagen IX in MED remains unclear, with conflicting reports on whether the mutant protein is retained within the endoplasmic reticulum or secreted into the ECM [21,22,24]. However, due to the remarkable clustering of all MED-collagen IX mutations in the COL3 domain, it is hypothesised that they may disrupt matrix structural integrity and affect ECM interactions, contributing to disease pathology.

Clinically, individuals with these mutations often present with normal to mild short stature, mild myopathy, and significant pain due to joint deformities and early-onset osteoarthritis, predominantly occurring in the knee [22,25]. While *COL9A2* mutations do not typically affect the hips, *COL9A3* mutations can cause significant hip involvement, demonstrating phenotypic heterogeneity depending on the specific disease-causing mutation [7,19,26,27].

**Table 2:** Collagen IX mutations causing multiple epiphyseal dysplasia.

Gene	Gene Location	Protein Change	Reference
*COL9A1*	IVS8 as + 3, ins A	Exon 8 and/or 10 skipping resulting in the in-frame deletion of 25/21/49 amino acids from the COL3 domain	[5]
*COL9A2*	IVS3 ds − 1, G > A	Exon 3 skipping resulting in the in-frame deletion of 12 amino acids from the COL3 domain	[25,28]
*COL9A2*	IVS3 ds − 1, G > C		[29]
*COL9A2*	IVS3 ds, G > A		[30]
*COL9A2*	IVS3 ds, G > C		[30]
*COL9A2*	IVS3 ds + 2, T > C		[25,26]
*COL9A2*	IVS3 ds + 4, A > C		[30]
*COL9A2*	IVS3 ds + 5, G > C		[28]
*COL9A2*	IVS3 ds + 6, T > G		[21]
*COL9A3*	IVS2 as − 1, G > A		[22,31]
*COL9A3*	IVS2 as − 2, A > T		[7]
*COL9A3*	IVS2 as − 2, A > G		[30]
*COL9A3*	IVS3 ds + 5, G > A		[27]
*COL9A3*	Exon 2, c.G104 > A	p.Gly35Asp missense mutation in the COL3 domain	[23]

### 2.2. Cartilage Oligomeric Matrix Protein

COMP, otherwise known as thrombospondin 5 (TSP5), is a non-collagenous calcium-binding glycoprotein predominantly found in cartilage localised within the chondrocyte territorial matrix [32,33]. COMP exists in the ECM as a disulphide-linked homopentamer consisting of five identical 524 kDa subunits. The pentameric structure is assembled through its N-terminal cysteine-rich coiled-coil domain, with each monomer comprising four epidermal growth factor (EGF)-like type II repeats, eight calmodulin-like type III (T3) repeats containing conserved Asparagine residues and calcium-binding domains, and a large C-terminal globular domain (CTD) [34].

The precise biological function of COMP is not fully understood, but it is believed to play a key role in regulating collagen secretion, fibril formation and organisation, and maintenance of the collagen network within the ECM [35,36]. Indeed, the CTD has been shown to interact with collagen family members (type I collagen, type II collagen, and type IX collagen), contributing to ECM stability [37,38,39]. Additionally, COMP interacts with a range of other ECM proteins, including fibronectin, proteoglycans, and growth factors, serving as a bridging molecule that likely regulates cellular differentiation and activity [40,41,42,43]. Furthermore, the presence of an RGD sequence within the T3 repeats suggests that COMP may also mediate cell binding via interactions with integrins [44].

The first mutations linked to PSACH and MED, including the most common and well-studied PSACH-causing p.Asp469del mutation (found in 30% of cases), were identified in 1995 by genetic mapping to a region on chromosome 19 (19p.13.1), which corresponded to the location of the *COMP* gene [3]. To date, >300 disease-causing *COMP* mutations have bene published (COMPopathies). These mutations typically impair calcium binding and protein folding, with 85% occurring within the T3 repeats and 15% in the CTD (Table 3). In addition, there is also some evidence that mutations in the exons encoding the type II repeats might also cause PSACH-MED-related phenotypes [30,45]. It is important to note that *Comp*-knockout mice do not exhibit skeletal abnormalities, while transgenic mutant mice display characteristics of skeletal dysplasia, confirming that the phenotype is not due to the lack of COMP in the matrix but rather to disrupted protein folding and homeostasis [46]. Interestingly, mutations affecting the same amino acid can result in either PSACH or MED. For example, the deletion of Asparagine at position 473 leads to more severe PSACH, whereas a duplication of Asparagine at position 473 causes the milder MED, which is attributed to the duplication being less disruptive to protein folding [30,47,48].

### 2.3. Matrilin-3

Matrilin-3 is the shortest and least complex member of the matrilin family of proteins, consisting of one von Willebrand factor A (vWFA) domain, four EGF-like domains, and a C-terminal α-helical coiled-coil domain [63]. The vWFA domain is known to facilitate protein–protein interactions, enabling the formation of multimeric complexes [64]. It is therefore hypothesised that matrilin-3 utilises its vWFA domain to mediate interactions with ECM proteins, thereby playing a role in maintaining cartilage structure. Specifically, due to its ability to bind collagens, proteoglycans, and non-collagenous ECM proteins such as COMP, matrilin-3 has been described as a molecular adaptor, bridging networks and coordinating the fibril network within the cartilage ECM.

In 2001, a study revealed that a diagnosis of MED in two unrelated families did not result from mutations in the genes encoding COMP or type IX collagen, highlighting the existence of a fourth MED locus [4]. Through genome linkage analysis and a candidate gene approach, *MATN3* on chromosome 2p24-p23 was chosen as a candidate gene due to its restricted expression in cartilage [63]. From this study, the first missense mutations, p.Arg121Trp and p.Val194Asp, were discovered [4]. EDM5 is the rarest form of MED, and since the discovery of MED as a causative gene, many more mutations in *MATN3* have been identified (Table 4), with the majority being missense mutations located in exon 2, which encodes the vWFA domain. Notably, ~70% of these mutations occur in structurally important conserved residues of the β-sheet within the vWFA domain and result in misfolding and intracellular accumulation of the misfolded protein [65,66]. To date, only a few mutations have been found outside of the vWFA domain, such as p.R70H, which is located just five amino acids away from the vWFA domain and is hypothesised to affect its folding [66,67]. While the majority of MED-causing *MATN3* mutations are missense mutations, an in-frame deletion/insertion resulting in a p.Asp171_Glu177delinsGlu mutation within the α4 helix of the vWFA domain has also been identified in one patient with EDM5, broadening the types of disease-causing matrilin-3 mutations [4].

## 3. Disease Mechanisms Determined in Mouse Models of PSACH-MED: ER Stress as a Common Denominator

**Figure 1:**
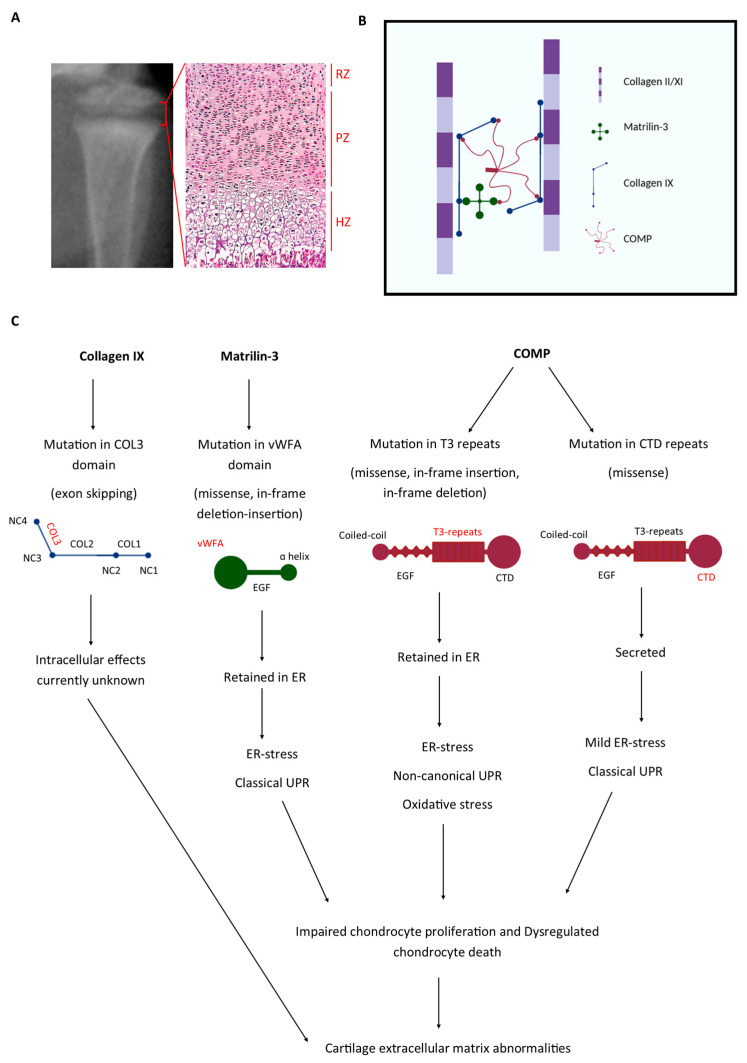
Disease mechanisms associated with PSACH-MED: COMP, matrilin-3, and collagen IX mutations. (**A**) The epiphyseal growth plate contains of a single cell type, chondrocytes, which are organised into distinct zones according to their differentiation status. (**B**) Proliferating chondrocytes secrete COMP, matrilin-3, and collagen IX, which interact to organise and stabilise the cartilage extracellular matrix. (**C**) PSACH-MED disease-causing mutations in these genes disrupt both intracellular homeostasis and cartilage extracellular matrix organisation. Exon-skipping mutations in the COL3 domain of collagen IX, currently being modelled in mice, are anticipated to compromise extracellular matrix assembly; however, their intracellular effects remain unknown. Matrilin-3 missense mutations within the vWFA domain lead to the intracellular retention of the mutant protein and the activation of a pathological UPR, with downstream effects on chondrocyte proliferation and apoptosis. In addition, loss of its linker function disrupts extracellular matrix organisation. COMP mutations have domain-specific effects: T3 mutations cause protein retention, triggering non-canonical ER stress and oxidative stress that disrupt both chondrocyte function and the extracellular matrix, whereas CTD mutations permit secretion of mutant COMP, triggering a mild UPR, with downstream consequences for intracellular function and matrix organisation. Overall, these mutations exert both intracellular and extracellular effects, highlighting their dual impact on chondrocyte biology and extracellular matrix integrity. (RZ = resting zone, PZ = proliferative zone, HZ = hypertrophic zone, COL = collagenous, NC = non-collagenous, vWFA = von Willebrand factor A, ER = endoplasmic reticulum, UPR = unfolded protein response, EGF = epidermal growth factor, T3 = Type III, and CTD = C-terminal domain).

To investigate the disease mechanisms of PSACH-MED in vivo, a series of ‘knock-in’ and transgenic mouse models were generated and studied in detail (Table 5 and Table 6). Although collagen IX-mutant mice have not been studied to date, mouse lines carrying the archetypal p.Asp469del COMP, p.Thr585Met COMP, and p.Val194Asp matrilin-3 PSACH-MED mutations have been successfully created [72,73,74]. These models have provided valuable insights into the role of cellular stresses in the pathological mechanisms underlying these diseases (Figure 1).

Most *COMP* mutations, particularly those in the T3 repeats, lead to the misfolding and intracellular retention of mutant COMP within the endoplasmic reticulum (ER), thereby causing ER stress. Interestingly, this retention of mutant COMP does not trigger a typical unfolded protein response (UPR). Instead, a novel ER stress response is activated, characterised by inflammation, oxidative stress, changes in cell cycle regulation, and dysregulated chondrocyte apoptosis [74]. The notable exception to this pattern is mutations in the C-terminal domain of COMP, which appear to allow the mutant protein to be secreted to some extent. Similarly, some mutations within the α-helical regions of the vWFA domain of matrilin-3 allow the mutant protein to be secreted; however, the majority of *MATN3* mutations result in the misfolding of the matrilin-3 protein, which, like most mutant forms of COMP, results in ER retention. In contrast to retained COMP, the ER stress induced by mutant matrilin-3 triggers a classic UPR.

Since *Matn3-* and *Comp*-null mice exhibited no skeletal abnormalities or clinical signs of PSACH-MED, this suggests that it is not the reduced expression of these ECM proteins but rather the expression of the mutant forms that drives the pathology. To confirm a direct link between ER stress and the PSACH-MED pathology, mouse lines were generated in which specific mutant forms of thyroglobulin (Tg) (p.Leu2269Pro: *Tg^co^*^g^ and p.G3230Arg: *Tg^rdw^*) were expressed in proliferating chondrocytes using the *Col2a1* promoter [79,80]. These mutations resulted in the retention of mutant Tg within the ER, causing chronic ER stress. One mutation led to a robust UPR (*Tg^co^*^g^), while the other triggered oxidative stress (*Tg^rdw^*). Both mouse models exhibited short-limbed dwarfism, confirming that ER stress induction alone can impair bone growth.

### Targeting ER Stress Is an Attractive Therapeutic Target in Genetic Bone Diseases

Recently, ER stress has been identified as a common ‘core’ disease mechanism in several rare bone diseases and in connective tissue diseases in general [81]. In these GSDs, the resulting UPR or oxidative stress response, triggered by ER stress, has detrimental downstream consequences on chondrocyte phenotype, function, and viability [82]. As a result, ER stress has emerged as an attractive therapeutic target for several GSDs and other rare diseases in general. This is best illustrated by the repurposing of carbamazepine (CBZ) for the treatment of metaphyseal chondrodysplasia type Schmid (MCDS), which is caused by mutations in type X collagen [83]. CBZ was shown to enhance the degradation of ER-retained mutant proteins by either autophagy or ER-associated protein degradation (ERAD) in a mutation-specific manner [83,84]. Based on these pre-clinical studies, CBZ was granted an orphan drug designation by the European Medicines Agency (EMA/623846/2016), and a clinical trial in children has just been completed. The MCDS-Therapy initiative underscores the potential of targeting ER stress as a therapeutic approach and emphasises the power of drug repurposing in the treatment of rare diseases.

Although CBZ has been shown as ineffective in the treatment of PSACH-MED, several studies offer promising alternative approaches. For example, research has demonstrated that the antioxidant resveratrol reduces the accumulation of mutant COMP through the stimulation of autophagy; a mechanism shared with CBZ in the treatment of MCDS [85]. In recent studies, curcumin has been shown to alleviate ER stress in a cell model of *MATN3*-MED (specifically the p.Val194Asp mutation), with its mode of action involving the enhanced proteolysis of the mutant matrilin-3 protein, again similar to the action of CBZ in MCDS [86]. Furthermore, CurQ+, a next-generation formulation of curcumin, has been shown to reduce chondrocyte stress and restore long bone growth in a transgenic COMP mouse model of PSACH [87].

As well as drug repurposing, pharmacological chaperones, which aim to reduce ER stress by enhancing protein folding and ER-associated degradation, have been explored as potential therapies for MED and PSACH. Although 4-phenylbutyrate (4-PBA) reduces pathological ER stress in a variety of diseases, it had no effect on matrilin-3 retention or aggregation, did not alleviate chondrocyte stress, and failed to improve the phenotype of p.V194D-mutant matrilin-3 mice. Similarly, the chemical chaperone tauroursodeoxycholic acid (TUDCA) had no effect in reducing ER stress in a cell model of matrilin-3 MED (Mularczyk et al.; unpublished data), highlighting the mutation-specific limitations of this approach.

To support the development of ER-stress–targeted therapies, it is important to establish biomarkers of disease activity and treatment response. ER-stress–related proteins could serve as indicators of chondrocyte stress and UPR activation, while circulating matrix proteins like COMP fragments may reflect cartilage turnover. Combining biochemical readouts that reflect cell stress and matrix turnover with advanced imaging techniques would enable comprehensive assessments of epiphyseal development and cartilage integrity, facilitating the monitoring of disease progression and therapeutic efficacy in preclinical and future clinical studies.

## 4. Changes in ECM Structure/Function in Cartilage in PSACH-MED

While the *Tg^cog^* and *Tg^rdw^* mouse models confirm that ER stress in proliferating chondrocytes leads to reduced bone growth and impaired chondrocyte proliferation, they do not recapitulate all the phenotypic features of PSACH-MED, such as dysregulated apoptosis, disrupted ECM architecture, and altered cell morphology [79,80]. This suggests that although ER stress contributes to the disease mechanism, the pathology of PSACH-MED results from a complex interplay of both intracellular factors (cell stress) and extracellular factors (ECM disruption).

Immunohistological analysis of the growth plate and articular cartilage from the PSACH-MED mouse models revealed that while the p.Asp469del COMP mutation and the p.Val194Asp matrilin-3 mutation cause the majority of the mutant protein to be retained within the ER, a small proportion of the mutant protein was found to be secreted into the ECM in mice homozygous for these mutations [72,73,74]. In contrast, the majority of p.T585M-mutant COMP was secreted into the ECM (Table 6) [78]. This observation of mutant structural proteins in the ECM therefore prompted further analysis into the impact of these mutant proteins on cartilage structure and function to better understand their role in the pathogenesis of PSACH-MED.

### 4.1. Understanding the Effects of Mutant Protein Expression in the Cartilage ECM in PSACH-MED

#### 4.1.1. Secretion of Mutant Matrilin-3 Disturbs the Collagen Networks in Growth Plate Cartilage

Immunohistochemistry revealed that secreted p.Val194Asp-mutant matrilin-3 was evenly distributed throughout the ECM, with no disruption to the localisation of key cartilage components, including matrilin-1, COMP, types II/IX/X collagen, and aggrecan [72,73]. Transmission electron microscopy analysis of the inter-territorial ECM from both proliferating and hypertrophic zones of the cartilage growth plate showed more pronounced collagens fibrils in the mutant cartilage [72]. This suggests that the ECM expression of mutant matrilin-3 results in a reduction in the levels of collagen fibril-associated proteins and glycoproteins, which may have a profound effect on the structure and/or function of the ECM.

#### 4.1.2. Secretion of Mutant p.T585M COMP Alters Its Localisation in Growth Plate Cartilage

COMP has a predominantly pericellular localisation at birth, but later it is found in the interterritorial matrix of the growth plate. In contrast, p.Thr585Met-mutant COMP was detected in greater abundance in the territorial and pericellular ECM of the growth plate cartilage, showing a notable difference from the uniform interterritorial distribution observed in wild-type mice [78]. Immunohistochemistry further revealed altered localisation of proteins known to interact with COMP. For example, matrilin-3 exhibited a more pronounced territorial and pericellular location, compared to its normal localisation within the interterritorial ECM. A similar pattern was noted for type IX collagen, whereas type II collagen remained unaffected. Transmission electron microscopy of the interterritorial matrix in the mutant mice showed a reduced presence of proteoglycan-like amorphous material, which resulted in more prominent collagen fibrils [79]. This could be attributed to the mislocalisation of type IX collagen and/or matrilin-3 in the ECM. Additionally, chondrocytes in the growth plates of p.Thr585Met-mutant COMP mice were severely misshapen and exhibited disrupted organisation [79], suggesting that the altered ECM contributed to these abnormal cellular changes.

#### 4.1.3. Secretion of Mutant p.Asp469del COMP Alters Localisation of Other Components of the ECM

The secretion of p.Asp469del-mutant COMP altered the appearance of the growth plate cartilage ECM, with type II collagen showing an uneven and diffuse distribution [74]. In contrast, the abundance of mutant COMP, matrilin-3, and collagen IX, to some extent, was significantly reduced, with both matrilin-3 and type IX collagen being co-retained in the ER alongside mutant COMP. Transmission electron microscopy revealed changes in the morphology of the ECM, particularly in the pericellular region of the growth plate [74]. These included more pronounced appearing collagen fibrils, consistent with a reduction in the levels of fibril-associated surface material such as COMP.

### 4.2. ER Stress or Matrix Integrity—Which Is the Driver of Osteoarthritis in PSACH-MED?

In PSACH and MED, the ER retention of mutant COMP or matrilin-3 in chondrocytes activates stress pathways that promote chondrocyte apoptosis and impair matrix integrity, hallmarks of osteoarthritic change. It remains unclear whether OA progression in PSACH-MED arises primarily from the loss of functional matrix proteins in the extracellular space, from toxic intracellular stress responses, or from a combination of both. In fact, these mechanisms likely converge with intracellular ER stress disrupting chondrocyte function and survival, and ECM protein deficiency and/or misassembly of matrix proteins, compromising their biomechanical stability. Furthermore, skeletal dysplasia-related joint shape abnormalities and ligament laxity increase the mechanical load, further accelerating degeneration. Thus, although the relative contribution of ER stress versus matrix deficiency remains unresolved, both are plausible drivers of the early-onset OA observed in these conditions.

## 5. Changes in ECM Structure/Function in Muscle, Ligaments, and Tendons in PSACH-MED

Although skeletal dysplasias are conditions affecting the growth and development of the skeleton, due to the complex interactions between bones, skeletal muscle, tendons, and ligaments, it comes as no surprise that abnormalities in one such biomechanical tissue can have a profound impact on others. Clinical findings have shown that neuromuscular abnormalities may manifest before the skeletal phenotype, thereby complicating diagnosis. Indeed, patients are often initially referred to neuropediatric clinics for symptoms of “non-defined” mild myopathy such as muscular hypotonia, difficulties with fine motor skills, and delayed walking only to be later diagnosed with chondrodysplasia [88]. While hypotonia becomes less prominent with age, other soft tissue abnormalities, such as ligament laxity and myopathy, can exacerbate or contribute to classical skeletal deformities including genu varum (bowlegs) and genu valgum (knock knees), as well as joint degeneration (osteoarthritis). As a result, many patients require joint-replacement surgery at an early age.

### 5.1. PSACH and MED Patients Present with Mild Myopathy

PSACH and MED were originally characterised by impaired bone growth, joint disruption, and ligament laxity; however, due to their genetic and clinical heterogeneity, these conditions are often difficult to diagnose. In addition to the diagnostic challenge, clinically variable myopathy symptoms have also been observed in PSACH-MED patients, particularly those with mutations in genes encoding type IX collagen, COMP, and, more recently, matrilin-3 (Table 7).

#### 5.1.1. Myopathy in EDM3

MED-causing *COL9A3* exon-skipping mutations were first found to be associated with a mild myopathy phenotype, characterised by proximal muscle weakness and mild to moderate elevations in serum creatine kinase (CK) levels, which is a marker of muscle damage [22]. In addition, muscle biopsies revealed a mild variability in muscle fibre size, further confirming the presence of a muscle disease in EDM3 patients.

Most recently *COL9A2* exon-skipping mutations in unrelated families have also been linked to mild myopathy, thereby expanding the spectrum of mutant genes involved in MED-associated muscle diseases [25]. Despite the established connection between type IX collagen mutations and myopathy with a neuromuscular phenotype, it is not a consistent feature in all cases of EDM3. It has been hypothesised that the clinical variability in muscle phenotype is likely related to the severity of the MED phenotype, with more severe skeletal involvement correlating with more pronounced myopathy.

#### 5.1.2. Myopathy in EDM1

Shortly after the initial discovery of muscle abnormalities in EDM3, mild myopathy was also reported in EDM1 patients with CTD COMP mutations [89]. Clinical findings in EDM1 patients included delayed motor development, which was attributed to joint laxity, muscle hypotonia; and mild myopathy, along with a slight increase in CK levels.

Several other CTD and even T3 COMP mutations associated with PSACH-MED have since been linked to a myopathic phenotype [49]. Patients with these mutations often report muscle weakness and fatigue, hypermobility, and trouble rising from the floor. Although serum CK levels were found to be within the normal range and a muscle biopsy from one such patient with a CTD COMP mutation (p.Asp605Asn) did not show any variation in muscle fibre diameter, it none the less did reveal the presence of scattered basophilic fibres and small atrophic fibres, both characteristic of mild myopathy [49].

#### 5.1.3. Myopathy in EDM5

Although several PSACH and MED patients with mutations in COMP and type IX collagen have reported muscular complications, to date only one patient, with a novel missense mutation in the vWFA domain of matrilin-3 p.Asp176Val, has presented with a suspected muscle disease [88]. As is the case with many PSACH-MED patients, this patient was initially incorrectly diagnosed as having a neuromuscular disorder due to fatigue following walking before being correctly diagnosed with MED.

### 5.2. Understanding the Mechanisms of PSACH-MED-Associated Myopathy

Despite extensive research focusing on the effects of PSACH-MED-mutant ECM proteins in cartilage development and homeostasis, their impact on the biomechanical musculoskeletal system remains poorly understood. The debilitating neuromuscular symptoms associate with these conditions can worsen the skeletal phenotype, thus highlighting the need to better understand the role of mutant cartilage ECM proteins in non-skeletal tissues. This new knowledge could significantly aid early diagnosis and improve clinical management, thereby ultimately enhancing patient quality of life.

To date, the association between mild myopathy and PSACH-MED remains inconclusive primarily due to the variability in neuromuscular clinical findings. Consequently, the use of mouse models has proven invaluable in deciphering disease mechanisms underlying the mild myopathy observed in PSACH and MED.

#### 5.2.1. Myopathy in Transgenic Knock-In Mouse Models of PSACH and MED

The muscle pathology associated with PSACH and MED has yet to be fully characterised. As such, we have generated the first mouse model to study the myopathy linked to MED, which is caused by mutant collagen IX (manuscript in preparation). To date, the p.Thr585Met CTD COMP-mutant knock-in mouse model has been generated and used to investigate PSACH-associated muscular, tendon, and ligament complications [79,90]. A detailed characterisation of the muscle phenotype in these mice revealed progressive myopathy primarily affecting the perimysium and the myotendinous junction, which are structures crucial for transmitting contractile forces from muscles to tendons [90]. For example, the collagen fibril architecture was disrupted, and there was an increased prevalence of fibres with central nuclei, which is a hallmark of muscle stress and remodelling in both tendons and ligaments of the mutant mice. Furthermore, the expression of p.Thr585Met-mutant COMP altered the biomechanical properties of tendons and ligaments, thereby leading to increased laxity and mild progressive muscle weakness, as demonstrated by a reduction in grip strength [90].

In addition to CTD COMP mutations, T3 COMP mutations have also been associated with mild myopathy, suggesting that the muscular complications are COMP mutation-specific and not domain-specific [49]. While the myopathy observed in the p.Asp469del COMP-mutant knock-in mouse model was less severe than that in the p.Thr585Met CTD COMP-mutant mice, they too displayed progressive muscle weakness due to an altered ultrastructure of collagen fibrils, along with an increase in the number of central nuclei in muscle fibres [90].

The finding that one MED patient with a matrilin-3 mutation presented with a neuromuscular disorder characterised by fatigue and difficulty walking suggested that matrilin-3 mutations could potentially contribute to muscle weakness, either directly through muscle/tendon pathology or indirectly due to short-limbed dwarfism [88]. Despite this finding, a mouse model harbouring the archetypal vWFA matrilin-3 mutation (p.Val194Asp) did not exhibit any muscle pathology [90]. Therefore, this highlights that muscle weakness and myopathy are associated with type IX collagen and COMP mutations but not with matrilin-3 mutations. This can be explained by the spatial localisation of these ECM proteins, because unlike COMP (expressed in muscles, tendons, and ligaments) [91] and collagen IX (expressed at sites where tendons or ligaments attach to bone) [92], matrilin-3 expression is restricted to cartilage [63]. Therefore, short-limbed dwarfism alone does not appear to contribute to muscle weakness; instead, the myopathy complications are likely a result of disrupted transmission of forces between tendon and muscle.

#### 5.2.2. Changes in Musculoskeletal ECM Structure/Function Due to the Expression of Mutant Type IX Collagen

To date, the role of mutant collagen IX in myopathy remains unclear. There is no evidence to suggest that collagen IX is expressed in skeletal muscle; rather, it is found at the fibrocartilage attachment site between tendon and bone, which is known as the enthesis [92]. Based on this understanding, it is therefore plausible to speculate that the muscle pathology stems from an underlying pathology at the enthesis, with the myopathy occurring as a secondary consequence of a tendinopathy. The resulting muscle weakness may therefore be attributed to impaired force transmission due to enthesial dysfunction. Additionally, the lack of significant myopathic changes in muscle biopsies from EDM3 patients further supports this theory since such changes would typically be expected if a muscle-specific structural protein was mutated.

#### 5.2.3. Changes in Musculoskeletal ECM Structure/Function Due to the Expression of Mutant COMP

Initially considered to be a cartilage-specific ECM protein, COMP has since been shown to be abundantly expressed in skeletal muscle, ligaments, and tendons [93]. In tendons COMP expression varies with age, mechanical loading, and muscle strength [91]. Specifically, COMP is expressed at low levels in tendons at birth, but its expression increases exponentially with age in weight-bearing tendons, thus suggesting that it plays a role in tendon growth and mechanical adaption [91]. Furthermore, COMP has been shown to localise in the matrix at sites with high concentrations of small fibrils, where it plays a critical role in reinforcing tendon structure and strength [94]. As a result, COMP levels correlate with the key biomechanical properties of tendons, including tensile strength, elasticity, and stiffness [95]. Research has also shown that in equine models, reduced COMP levels in tendons are linked to an increased risk of injury with tendinopathies associated with matrix breakdown and the subsequent loss of COMP [95].

To investigate the pathological mechanisms underlaying myopathy in PSACH-MED, a transgenic mouse model harbouring a CTD COMP mutation (p.Thr585Met), which results in a secreted form of mutant COMP, was generated and characterised in detail [79,90]. While COMP is expressed throughout muscle and tendons, the myopathy in these mice was localised to the perimysium and the myotendinous junction and was accompanied by a generalised tendinopathy [90]. Given that the muscle pathology was restricted to areas where the muscle attaches to the tendon and transmits force, it was hypothesised that the myopathy arises from impaired force transmission due to tendon dysfunction. This finding provides insight as to how mutations in type IX collagen, which is expressed in the fibrocartilage at the tendon–bone interface, may also contribute to myopathy, as these mutations likely disrupt force transmission rather than directly affecting the tendon structure.

In addition to CTD mutations, although T3 mutant COMP is often retained in chondrocytes, it can be secreted in tendons and ligaments [96,97] causing a myopathy. 

Since *Comp*-knockout mice exhibit normal tendon morphology, this suggests that the myopathy in PSACH-MED arises specifically from the secretion of mutant protein within the ECM [46]. Given that force transmission requires a strong connection between tendon and muscle, it is hypothesised that the myopathy in PSACH-MED is a direct consequence of impaired force transmission and is driven by alterations in the ECM architecture due to the presence of mutant COMP in the matrix. Notably, not all CTD and T3 COMP mutations cause myopathy, prompting the suggestion that the muscle pathology may be linked to the proximity of the mutation to potential bindings sites within COMP [49,88]. For example, COMP has been shown to bind to integrins [44], which are implicated with myopathy, suggesting that disrupting the ECM may contribute to the muscle pathologies observed in PSACH-MED.

## 6. Conclusions

Mouse models have been instrumental in elucidating PSACH-MED disease mechanisms and have demonstrated that ER stress is a key driver of pathology. However, this review highlights that disease progression is further complicated by 1. the absence of functional matrix protein or 2. the secretion of mutant protein into the ECM. Both situations result in ECM disorganisation as shown by matrix protein mislocalisation, impaired collagen fibril formation, and reduced proteoglycan content that ultimately weakens the cartilage structure and contributes to biomechanical weakness and progressive joint degeneration. Interestingly, these ECM abnormalities extend beyond cartilage to soft tissues such as tendons, ligaments, and muscle attachments, disrupting their composition and function and leading to joint laxity, impaired force transmission, and further musculoskeletal dysfunction.

Although ER stress has emerged as a promising therapeutic target, with compounds such as carbamazepine, resveratrol, and curcumin showing potential in alleviating pathological cellular stress, the underlying ECM defects remain a challenge. A comprehensive understanding of ECM abnormalities in these conditions is therefore essential for designing interventions that go beyond cellular stress relief to reinforce the biomechanical stability of cartilage and associated musculoskeletal tissues. Advancing these strategies could provide meaningful therapeutic options for patients with PSACH, MED and other ECM-related skeletal disorders, ultimately improving clinical outcomes and quality of life.

## Figures and Tables

**Table 1:** Disease-causing mutations in ECM proteins that result in PSACH and MED.

Disease	Gene	Protein	Type of Mutation
MED (EDM6, OMIM #614135)	*COL9A1*	Type IX Collagen	Exon skipping and in-frame deletions
MED (EDM2, OMIM #600204)	*COL9A2*		
MED (EDM3, OMIM #600969)	*COL9A3*		
MED (EDM5, OMIM #607078)	*MATN3*	Matrilin-3	Antimorphic missense mutations and in-frame deletions
MED (EDM1, OMIM #134200)	*COMP*	Cartilage Oligomeric Matrix Protein	
PSACH (OMIM #177170)	*COMP*		

**Table 3:** COMP mutations causing pseudoachondroplasia and multiple epiphyseal dysplasia.

Mutation	Location	Disease	Reference
p.Asn555Lys	CTD	EDM1	[49]
p.Thr585Arg	CTD	PSACH/EDM1	[30]
p.Thr585Met	CTD	PSACH/EDM1	[5,50,51]
p.Thr585Lys	CTD	PSACH/EDM1	[30]
p.Thr585Arg	CTD	PSACH/EDM1	[30]
p.His587Arg	CTD	PSACH	[51,52]
p.Asp605Asn	CTD	EDM1	[51]
p.Ser681Cys	CTD	EDM1	[51]
p.Arg718Pro	CTD	EDM1	[51,53]
p.Arg718Trp	CTD	EDM1	[48,51,53,54]
p.Gly719Arg	CTD	PSACH	[55]
p.Gly719Ser	CTD	PSACH	[30,51]
p.Pro276Arg	T3	EDM1	[5]
p.Asp290Gly	T3	PSACH	[30]
p.Ser298Leu	T3	EDM1	[48]
p.Asp299Arg	T3	PSACH	[30]
p.Ser298Leu	T3	PSACH	[53]
p.Ala311Asp	T3	EDM1	[30]
p.Asp316Tyr	T3	EDM1	[56]
p.Asp317Gly	T3	EDM1	[30]
p.Asp326Tyr	T3	PSACH	[30,57]
p.Asp326Gly	T3	EDM1	[30]
p.Glu341_Asp342del	T3	PSACH	[30]
p.Cys348Phe	T3	EDM1	[30]
p.Asn350_Asp372del	T3	PSACH	[30]
p.Cys371Ser	T3	EDM1	[30,48]
p.Cys371Tyr	T3	EDM1	[58]
p.Asp374Asn	T3	EDM1	[48]
p.Asp376Asn	T3	EDM1	[48]
p.Asp378Val	T3	PSACH	[30]
p.Asp385Asn	T3	EDM1	[48]
p.Asp385del	T3	EDM1	[53]
p.Asp385Tyr	T3	EDM1	[30]
p.Cys387Arg	T3	PSACH	[30]
p.Asp397His	T3	EDM1	[30]
p.Asp401Asn	T3	EDM1	[48]
p.Gly404Arg	T3	EDM1	[30]
p.Cys407Arg	T3	EDM1	[59]
p.Cys410Tyr	T3	PSACH/EDM1	[48]
p.Asn415Lys	T3	EDM1	[48]
p.Gly427Glu	T3	EDM1	[48,52]
p.Asp439Glu	T3	PSACH	[60]
p.Gly440Arg	T3	PSACH	[30]
p.Asp446Asn	T3	PSACH	[30]
p.Cys448Ser	T3	PSACH	[30]
p.Glu457del	T3	EDM1	[54,61]
p.Asp469-Asp473del	T3	PSACH	[51]
p.Asp473dup	T3	EDM1	[47]
p.Asp473del	T3	PSACH	[30]
p.Asp473His	T3	PSACH	[30]
p.Asp473Tyr	T3	PSACH	[51]
p.Asp473_Asp474insAsp	T3	EDM1	[48]
p.Asp475Asn	T3	PSACH	[30]
p.Asp482Gly	T3	PSACH	[30]
p.Asp482His	T3	PSACH	[51]
p.Gly501Asp	T3	EDM1	[30]
p.Gly501Asp_Asp502Tyr	T3	EDM1	[30]
p.Asp507Gly	T3	PSACH	[30]
p.Asp511Gly	T3	PSACH	[30]
p.Asp515Gly	T3	PSACH	[30]
p.Asn523Lys	T3	EDM1	[48,62]
p.Thr529Ile	T3	PSACH	[30]
p.Thr529Ala	T3	PSACH	[57]
p.Thr529Ile	T3	PSACH	[30]
p.Thr529Ala	T3	PSACH	[57]

**Table 4:** Matrilin-3 mutations causing multiple epiphyseal dysplasia.

Mutation	Location	Reference
p.Arg70His	Linker region	[67]
p.Phe105Ser	A domain: α1	[68]
p.Thr120Met	A domain: βB	[65,68]
p.Arg121Trp	A domain: βB	[4,65,66,68]
p.Ala123Lys	A domain: βB	[68]
p.Ala128Pro	A domain: βB	[69]
p.Glu134Lys	A domain: βC	[65]
p.Asp171_Glu177delinsGlu	A domain: α4	[30]
p.Ala173Asp	A domain: α4	[70]
p.Ala191Asp	A domain: βD	[71]
p.Ile192Asn	A domain: βD	[65]
p.Val194Asp	A domain: βD	[4]
p.Thr195Lys	A domain: βD	[66]
p.Arg209Pro	A domain: α5	[30]
p.Tyr218Asn	A domain: βD	[66]
p.Ala219Asp	A domain: βE	[65]
p.Lys231Asn	A domain: α6	[70]
p.Val245Met	A domain: βF	[30]

**Table 5:** Mouse models of the PSACH-MED disease spectrum and novel phenocopies to model ER stress in the cartilage growth plate.

Disease	Gene	Mutation	Approach Taken	Promoter	Reference
PSACH	*COMP*	p.Asp469del	Transgenic (rat COMP cDNA)	*Col2a1*	[75]
PSACH	*COMP*	p.Asp469del	Transgenic (human COMP gene)	Native	[76]
PSACH	*COMP*	p.Asp469del	Transgenic (human COMP cDNA)	*Col2a1*	[76]
PSACH	*COMP*	p.Asp469del	Transgenic inducible overexpression (human COMP cDNA)	*Col2a1* and tetracycline responsive element	[77]
PSACH	*COMP*	p.Asp469del	Knock-in	Native	[74]
PSACH-MED	*COMP*	p.Thr585Met	Knock-in	Native	[78]
MED	*MATN3*	p.Val194Asp	Knock-in	Native	[72,73]
Chondrodysplasia	*TG*	*Rdw* (p.Gly2320Arg)	Transgenic ‘ER-stress phenocopy’	*Col2a1*	[79]
Chondrodysplasia	*TG*	*Cog* (p.Lys2293Pro)	Transgenic ‘ER-stress phenocopy’	*Col2a1*	[80]

**Table 6:** In vivo disease mechanisms associated with PSACH-MED: COMP and matrilin-3 mutations.

Gene	Mutation	Phenotype	Retained	ER Stress	Response to ER Stress	Reference
*COMP*	p.Asp469del	PSACH	Yes	Yes	Novel stress pathways	[74]
*COMP*	p.Thr585Met	Mild PSACH/MED	Slightly	Mild	Mild UPR	[78]
*MATN3*	p.Val194Asp	MED	Yes	Yes	UPR	[72,73]

**Table 7:** PSACH-MED gene mutations associated with myopathy.

Gene	Mutation	Clinical Feature	Diagnosis	Reference
*COL9A2*	Exon 3 Skipping IVS3 ds, G > C	Muscle weakness Difficulty rising from sitting	MED	ESDN-01013 [88]
*COL9A2*	Exon 3 skipping IVS3 ds + 4, A > C	Muscle weakness	MED	ESDN-01003 [88]
*COL9A2*	Exon 3 Skipping IVS3 ds + 2, T > C	Muscle weakness Difficulty rising from sitting and walking Muscle biopsy—variation in fibre size Easily fatigued ATP and creatine phosphate levels decreased	MED	ESDN-00997 [25]
*COL9A2*	Exon 3 skipping IVS2 ds − 1, G > A	Muscle weakness Difficulty walking	MED	ESDN-01013 [25]
*COL9A3*	Exon skipping IVS2 as − 1, G > A	Muscle weakness—referred to a neuromuscular unit Difficulty rising from sitting, walking, and climbing stairs Muscle biopsy—variation in fibre size Mildly elevated serum creatine kinase levels indicating muscle damage	MED	[22]
*COMP*	T3 domain mutation p.Asp326Tyr	Muscle weakness Difficulty walking and climbing stairs Mild myopathy	PSACH	ESDN-00385
*COMP*	T3 domain mutation p.Glu457del	Muscle weakness—diagnosed with myopathy Muscle biopsy—inconclusive	MED	ESDN-00430 [54,61]
*COMP*	CTD mutation p.Asp605Asn	Muscle weakness Difficulty rising from sitting Muscle biopsy—mild myopathy with scattered basophilic fibres and small atrophic fibres but no change in fibre size Normal creatine kinase levels NEasily fatigued	MED	[49]
*COMP*	CTD mutation p.Arg718Trp	Muscle weakness—referred to a neuromuscular clinic Mildly elevated creatine kinase levels	MED	ESDN-00066 ESDN-00080 [49,89]
*MATN3*	p.Asp176Val	Difficulty walking Easily fatigued	MED	ESDN-01071 [88]

## Data Availability

No new data were created or analysed in this study. Data sharing is not applicable to this article.

## Abbreviations

The following abbreviations are used in this manuscript:

CBZ	Carbamazepine
CK	Creatine kinase
COL	Collagenous
COMP	Cartilage oligomeric matrix protein
CTD	C-terminal domain
ECM	Extracellular matrix
EGF	Epidermal growth factor
ER	Endoplasmic reticulum
ERAD	ER-assisted protein degradation
FACIT	Fibril-associated collagens with interrupted triple helices
GSD	Genetic skeletal disease
MCDS	Metaphyseal chondrodysplasia type Schmid
MED	Multiple epiphyseal dysplasia
NC	Non-collagenous
PSACH	Pseudoachondroplasia
T3	Type III
Tg	Thyroglobulin
TSP5	Thrombospondin 5
UPR	Unfolded protein response
vWFA	Von Willebrand factor A
